# Comparison of macrophage migration inhibitory factor and neutrophil gelatinase-associated lipocalin-2 to predict acute kidney injury after liver transplantation: An observational pilot study

**DOI:** 10.1371/journal.pone.0183162

**Published:** 2017-08-15

**Authors:** Joanna Baron-Stefaniak, Judith Schiefer, Edmund J. Miller, Gabriela A. Berlakovich, David M. Baron, Peter Faybik

**Affiliations:** 1 Department of Anaesthesia, Intensive Care Medicine and Pain Medicine, Medical University of Vienna, Vienna, Austria; 2 The Feinstein Institute for Medical Research, Northwell Health System, Manhasset, New York, United States of America; 3 Department of Surgery, Division of Transplantation, Medical University of Vienna, Vienna, Austria; University of Sao Paulo Medical School, BRAZIL

## Abstract

**Introduction:**

Several biomarkers have been suggested as early predictors of acute kidney injury (AKI) after orthotopic liver transplantation (OLT). Neutrophil gelatinase-associated lipocalin-2 (NGAL) appears to be a promising predictor of AKI after OLT, but the clinical benefit remains to be proven. Recently, systemic macrophage migration inhibitory factor (MIF) has been proposed as early indicator for requirement of renal replacement therapy after OLT. The aim of this prospective, observational pilot study was to compare the predictive values of serum and urinary MIF for severe AKI after OLT to those of serum and urinary NGAL.

**Methods:**

Concentrations of MIF and NGAL were measured in serum and urine samples collected from patients undergoing OLT. Acute kidney injury was classified according to the KDIGO criteria, with stages 2 and 3 summarized as severe AKI. Areas under the receiver operating curves (AUC) were calculated to assess predictive values of MIF and NGAL for the development of severe AKI.

**Results:**

Forty-five patients (mean age 55±8 years) were included. Nineteen patients (38%) developed severe AKI within 48 hours after reperfusion. At the end of OLT, serum MIF was predictive of severe AKI (AUC 0.73; 95% confidence intervals, CI 0.55–0.90; P = 0.03), whereas urinary MIF, serum NGAL, and urinary NGAL were not. On the first postoperative day, serum MIF (AUC 0.78; CI 0.62–0.93; P = 0.006), urinary MIF (AUC 0.71; CI 0.53–0.88; P = 0.03), and urinary NGAL (AUC 0.79; CI 0.64–0.93; P = 0.02) were predictive for severe AKI, while serum NGAL was not.

**Conclusion:**

In the setting of OLT, MIF and NGAL had similar predictive values for the development of severe AKI.

## Introduction

Acute kidney injury (AKI) is a major complication after orthotropic liver transplantation (OLT) associated with increased morbidity and mortality, reduced graft survival, and prolonged hospital length-of-stay [1–3]. Up to 50% of patients undergoing OLT develop AKI [1,4–7]. Approximately 30% of patients with AKI require renal replacement therapy (RRT) following OLT [3,7,8]. Current management of AKI includes optimization of the fluid status and dose-adjustment of nephrotoxic drugs. Recent studies suggest that early institution of RRT may improve survival in patients with severe AKI [9,10], emphasizing the need for early recognition of postoperative AKI.

Acute kidney injury is diagnosed by evaluating changes in serum creatinine (sCr) concentrations and urine output [11]. Serum creatinine is a poor marker of AKI in patients with end-stage liver disease, as sCr concentrations may be influenced by decreased creatinine biosynthesis, reduced muscle mass, and increased serum bilirubin concentrations [12]. Additionally, sCr concentrations increase when renal injury is already present, limiting their use for early detection of AKI [13]. Systemic and urinary neutrophil gelatinase-associated lipocalin-2 (NGAL) have been suggested as promising biomarkers for early prediction of AKI in patients undergoing OLT [4,14–16]. However, as the clinical value of NGAL remains to be proven, new molecules are being investigated for their potential to predict AKI after OLT.

Recently, the pro-inflammatory cytokine macrophage migration inhibitory factor (MIF) has been associated with AKI in humans. Plasma MIF concentrations were independently associated with the severity of AKI in critically ill patients [17]. Furthermore, we have reported that systemic MIF has a good prognostic value to identify patients requiring postoperative RRT after OLT [7]. In addition, urinary MIF has been proposed as a predictor of AKI, particularly in patients with inflammatory nephritis [18,19] and kidney transplant rejection [20]. However, whether systemic or urinary MIF can predict the development of AKI after OLT with comparable power as systemic and urinary NGAL remains to be elucidated. In this current study we compared the predictive values of serum and urinary MIF for the development of severe AKI after OLT to those of serum and urinary NGAL.

## Patients and methods

### Study subjects

This single-center, prospective, observational pilot study was performed in accordance with the ethical standards laid down in the Declaration of Helsinki and the Declaration of Istanbul. Institutional ethics committee approval for the study was obtained (Ethikkommission der Medizinischen Universität Wien, reference number 1271/2014), and the study was registered at clinicaltrials.gov (NCT02695979). None of the transplant donors were from a vulnerable population. All patients provided written informed consent before inclusion.

Consecutive patients with end-stage liver disease scheduled for OLT at the General Hospital of Vienna between August 2014 and August 2015 were enrolled in the study. Preoperative kidney function was assessed by evaluating sCr concentrations measured at hospital admission immediately before OLT. Estimated glomerular filtration rate (eGFR) was calculated according to the ‘Modification of Diet in Renal Disease Study’ Equation [21]. Patients with severe preoperative kidney dysfunction (eGFR<30 mL/min/1.73 m^2^) were not enrolled in the study. Other exclusion criteria were combined liver-lung or liver-kidney transplantation, high urgency transplantation, and the requirement of veno-venous bypass during OLT.

### Anesthesia, surgery, and immunosuppression

Orthotopic liver transplantations were performed under general anesthesia using the local standard technique with cross-clamping of the caval vein. After transplantation, all patients were admitted to the intensive care unit (ICU). Immunosuppression with intravenous application of 40 mg dexamethasone was initiated before graft reperfusion. The second dose of intravenous dexamethasone (32 mg) was administered 24 hours after reperfusion, and was further reduced daily by 8 mg until reaching the maintenance dose of 8 mg. Additionally, postoperative immunosuppression was induced with anti-thymocyte globulin (2.5 kg per kg body weight) within two hours after arrival at the ICU and maintained with low-dose tacrolimus starting on the forth postoperative day (trough level 6–8 ng/ml).

### Data and sample collection

Epidemiological patient data were collected prior to surgery. The model for end-stage liver disease (MELD) score was calculated preoperatively to assess the severity of liver disease. Perioperative data including laboratory values, duration of surgery, cold ischemia time, caval clamping time, blood loss, transfusion of packed red blood cells, platelets and fresh frozen plasma, administration of coagulation factors, fluid balance, and urine output were recorded for one week following OLT. Data were extracted from the patient data management system (ICCA, Phillips Healthcare, Hamburg, GER). Serum and urine samples were collected at the following time points: at baseline (BL) during stable hemodynamic conditions after induction of anesthesia, prior to surgical skin incision; at the end of surgery (day 0); at 24 hours after reperfusion on the first postoperative day (day 1); and at 48 hours after reperfusion on the second postoperative day (day 2). Serum samples were sent to the central laboratory for analysis of blood chemistry, including sCr. For measurement of MIF and NGAL concentrations, serum and urine samples were centrifuged for 30 minutes at 1000 *g* and supernatants were stored at -80°C until analysis.

### Diagnosis and classification of AKI

Acute kidney injury was diagnosed and classified according to the KDIGO criteria (stage 0, 1, 2 and 3 AKI) [11]. Serum creatinine concentrations obtained from samples collected 48 hours after reperfusion (day 2) were compared to pre-operative sCr concentrations to diagnose AKI and classify the AKI stage. Patients met the criteria for stage 0 AKI (i.e. no AKI) if differences between pre-operative sCr concentrations and sCr concentrations on day 2 were below 0.3 mg/dl. Criteria for stage 1 AKI were met when sCr concentrations on day 2 were at least 0.3 mg/dl greater than pre-operative sCr concentrations. A 2-fold to 3-fold increase of sCr concentrations on day 2 from pre-operative sCr concentrations characterized stage 2 AKI. Criteria for stage 3 AKI were met when the sCr concentrations on day 2 were at least 3-fold compared to pre-operative sCr concentrations, or when sCr was above 4 mg/dl. Individuals receiving RRT within 48 hours after reperfusion were considered to have met the criteria for stage 3 AKI. After AKI classification, patients were separated into two groups: Patients developing no AKI or stage 1 AKI at day 2 after OLT were summarized as the no/mild AKI group, whereas patients meeting the criteria for stage 2 or 3 AKI at day 2 after OLT were summarized as the severe AKI group.

### MIF and NGAL concentrations and prediction of AKI

Enzyme-linked immunosorbent assays were used according to the manufacturer’s protocol to determine concentrations of MIF (human MIF Quantikine kit; R&D Systems, Minneapolis, MN) and NGAL (human Lipocalin-2 ELISA; RayBiotech, Norcross, GA) in serum and urine samples collected at baseline, day 0, day 1 and at day 2.

In order to assess whether MIF and NGAL could predict AKI prior to to conventional diagnosis of AKI using the KDIGO criteria, the predictive performance of MIF and NGAL was analyzed separately for day 0 and day 1. The discriminatory power to predict severe AKI was analyzed for serum MIF, serum NGAL, urinary MIF, and urinary NGAL, and compared between patients in the no/mild AKI group and those in the severe AKI group. In addition, the predictive power of MIF and NGAL for the development of any AKI was assessed by comparing values between patients not developing AKI and those developing any stage of AKI. Furthermore, we assessed the performance of MIF and NGAL to diagnose the development of AKI on day 2, which was the time point used to diagnose AKI by the KDIGO criteria.

### Statistical analysis

Statistical analyses were performed using Prism 6.0 (GraphPad Software, La Jolla, CA). Results are depicted as median with interquartile ranges (IQR 25%-75%), while continuous variables are expressed as mean ± standard deviation. Two-way ANOVA with Bonferroni correction was used to compare differences among groups at various time points. Differences within groups at various time points were analyzed with an unpaired t-test and corrected for multiple comparisons with the Holm-Sidak method. In order to determine the true positive rate in function of the false positive rate at different cut-off points, a receiver operating characteristics (ROC) curve analysis was performed for MIF and NGAL in serum and in urine as predictors of severe AKI. An area under the curve (AUC) of 0.90–1.0 indicated excellent, 0.80–0.89 good, 0.70–0.79 fair, 0.60–0.69 poor, and 0.50–0.59 no useful predictive value for the development of AKI [22]. For all statistical analyses, an adjusted P-value <0.05 was considered significant. Adjusted P values are reported when data have been corrected for multiple comparisons.

Sample size was calculated based on data from our previous study [7]. Calculation for an area under ROC curve of 0.75 with type I error of 0.05 and type II error of 0.2 (power 80%) estimated a sample size of 45 patients.

## Results

### Patient characteristics and incidence of AKI after OLT

Forty-five patients were enrolled in the study. Demographic data of the study population and the etiology of liver disease are listed in Table 1. On day 2 after OLT, 15 patients (33%) met the criteria for stage 1 AKI, 9 patients (20%) were diagnosed stage 2 AKI, and 10 patients (22%) met the criteria for stage 3 AKI. There were no differences in preoperative sCr concentrations, glomerular filtration rate, body-mass index, MELD score, or intraoperative transfusion requirements among the no/mild AKI group and the severe AKI group (Table 2). There was no difference in sCr concentrations between patients without severe AKI and those with severe AKI on day 0. As sCr was used to diagnose AKI, sCr concentrations were greater in patients developing severe AKI than in patients without AKI on day 1 and day 2 after OLT (“S1 Fig”). Furthermore, cold ischemia time and caval clamping time did not differ among groups. No major complications such as massive postoperative hemorrhage (requirement of >4 units of packed red blood cells over 48 hours), sepsis, hepatic artery occlusion, and caval vein thrombosis were observed in any patient during the ICU stay.

**Table 1:** Demographic data of the study population.

Sex	
Male, n (%)	36 (80)
Female, n (%)	9 (20)
Ethnicity	European
Etiology of end-stage liver disease	
Alcohol-induced, n (%)	22 (49)
Viral, n (%)	9 (20)
Hepatocellular carcinoma, n (%)	4 (9)
Autoimmune, n (%)	4 (9)
Primary biliary cirrhosis, n (%)	2 (4)
Other, n (%)	4 (9)

**Table 2:** Perioperative characteristics of patients undergoing OLT.

	All patients	No/mild AKI	Severe AKI	P value
Patients (n)	45	26	19	
Age (years)	55±9	54±8	56±10	0.37
MELD	17±6	16±4	18±7	0.23
eGFR (mL/min/1.73 m^2^)	96±37	85±32	111±39	0.42
Preoperative sCr (mg/dl)	0.9±0.3	1.0±0.3	0.8±0.3	0.41
Body-mass index	26±5	27±4	25±5	0.45
Cold ischemia time (min)	345±148	354±186	332±60	0.68
Caval clamping time (min)	88±22	88±26	88±16	0.99
PRBC units transfused, n	3±3	3±3	4±4	0.14
FFP units transfused, n	6±6	6±6	7±7	0.46
Thrombocyte units transfused, n	1±1	1±1	1±1	0.75

Data are depicted as mean ± standard deviation. P values indicate differences among the no/mild AKI and the severe AKI group. Abbreviations: AKI, acute kidney injury; eGFR, estimated glomerular filtration rate; FFP, fresh frozen plasma; MELD, model for end stage liver disease; PRBC, packed red blood cell; sCr, serum creatinine.

### Serum concentrations of MIF and NGAL in patients with severe AKI

**Fig 1:**
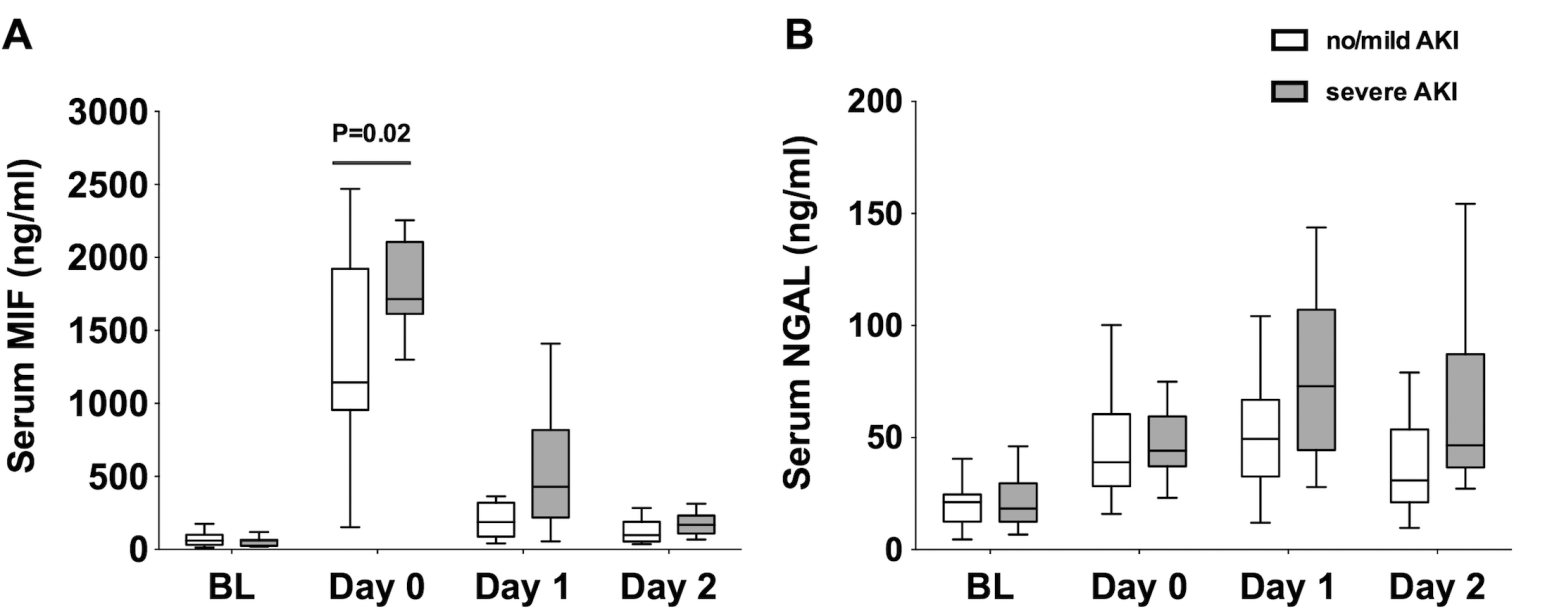
Serum concentrations of MIF and NGAL. Concentration of (A) serum MIF and (B) serum NGAL at 4 different time points: baseline (BL; under anesthesia before skin incision), day 0 (at the end of surgery), day 1 (24 hours after graft reperfusion on day 1 after OLT), and on day 2 (48 hours after reperfusion on day 2 after OLT). White bars indicate values of patients with no AKI or stage 1 AKI, gray bars represent values of patients who developed stage 2 or 3 AKI after undergoing OLT. P values indicate significant differences between groups.

Baseline serum MIF concentrations did not differ among the no/mild AKI group (60 ng/ml, IQR 31–100) and the severe AKI group (60 ng/ml, IQR 27–66, P = 0.90; “Fig 1A”). At the end of surgery (day 0), serum MIF concentrations increased in both groups (P<0.001), but were lower in the no/mild AKI group (1143 ng/ml, IQR 955–1922) than in the severe AKI group (1715 ng/ml, IQR 1613–2106, P = 0.02). At day 1 after OLT, serum MIF concentrations returned to baseline values in the no/mild AKI group (187 ng/ml, IQR 87–320, P = 0.50 vs. baseline), but remained elevated in the severe AKI group (429 ng/ml, IQR 218–818, P = 0.007 vs. baseline). At day 2 after OLT, serum MIF concentrations did not differ from baseline values in the no/mild AKI group (98 ng/ml, IQR 54–189, P = 0.76 vs. baseline) or in the severe AKI group (169 ng/ml, IQR 109–232, P = 0.28 vs. baseline).

Serum NGAL concentrations at baseline did not differ among the no/mild AKI group (21 ng/ml, IQR 13–25) and the severe AKI group (18 ng/ml, IQR 12–30, P>0.99; “Fig 1B”). At the end of OLT (day 0), serum NGAL concentrations did not differ from baseline in the no/mild AKI group (39 ng/ml, IQR 28–60, P = 0.08) or in the severe AKI group (44 ng/ml, IQR 37–59, P = 0.10), and did not differ among groups (P>0.99). At day 1 after OLT, serum NGAL concentrations increased to 49 ng/ml, IQR 33–67 in the no/mild AKI group (P = 0.005) and to 73 ng/ml, IQR 44–107 in the severe AKI group (P<0.001), but did not differ among groups (P = 0.73). At day 2 after OLT, serum NGAL concentrations returned to baseline values in the no/mild AKI group (31 ng/ml, IQR 21–54, P = 0.44), but remained elevated in the severe AKI group (47 ng/ml, IQR 37–87 P = 0.004 vs. baseline).

### Urinary concentrations of MIF and NGAL in patients with severe AKI

**Fig 2:**
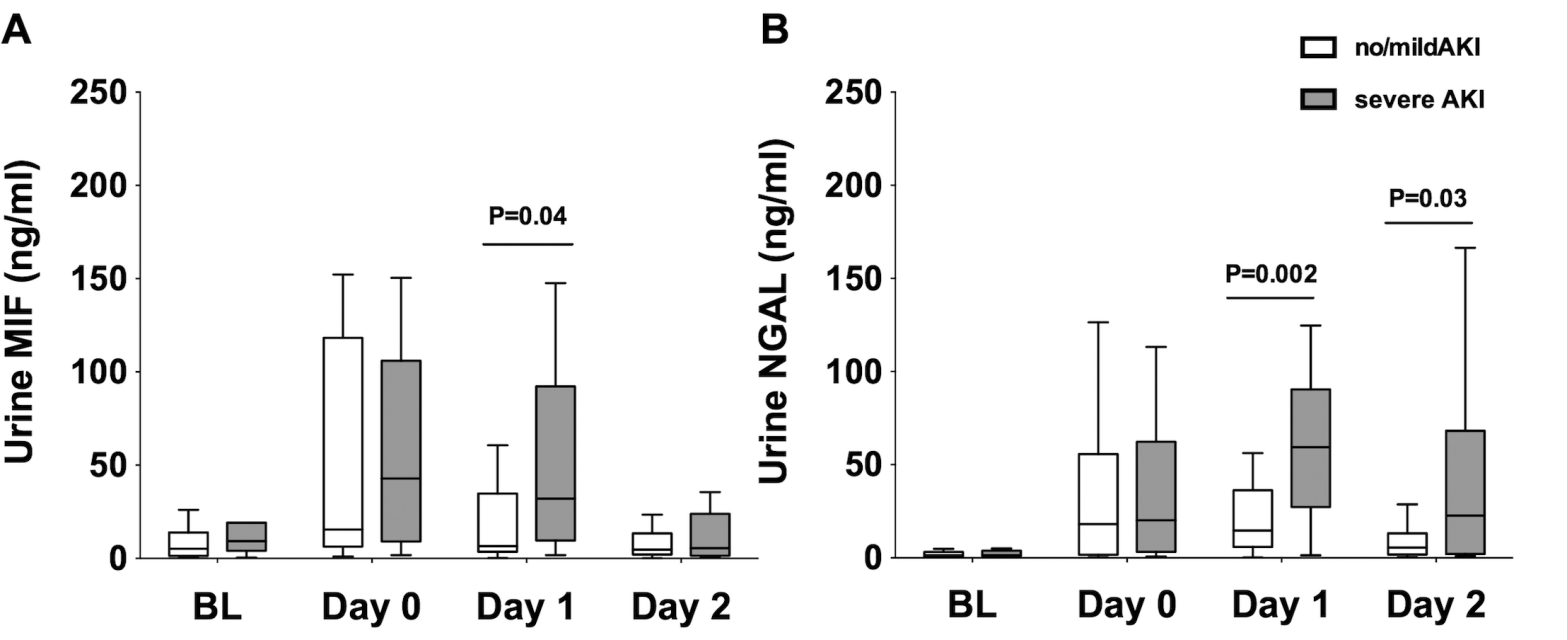
Urine concentrations of MIF and NGAL. Concentrations of (A) urinary MIF and (B) urinary NGAL at 4 different time points: baseline (BL; under anesthesia before skin incision), day 0 (at the end of surgery), day 1 (24 hours after graft reperfusion on day 1 after OLT), and on day 2 (48 hours after reperfusion day 2 after OLT). White bars indicate values of patients with no AKI or stage 1 AKI, gray bars represent values of patients who developed stage 2 or 3 AKI after undergoing OLT. P values indicate significant differences between groups.

Urine MIF concentrations at baseline did not differ among patients with no/mild AKI (5 ng/ml, IQR 1.4–14) and those with severe AKI (9 ng/ml, IQR 4–19, P = 0.87; “Fig 2A”). At the end of OLT (day 0), urine MIF concentrations increased to 15 ng/ml, IQR [6–118] in the no/mild AKI group (P = 0.003) and to 43 ng/ml, IQR 9–106 in the severe AKI group (P = 0.02), but did not differ among groups (P = 0.95). At day 1, urine MIF concentrations were lower in the no/mild AKI group (6.5 ng/ml, IQR 4–35) than in the severe AKI group (32 ng/ml, IQR 9.5–92, P = 0.04). At day 2 after OLT, urine MIF concentrations returned to baseline values in both groups.

Urinary NGAL concentrations at baseline did not differ among patients with no/mild AKI (1.7 ng/ml, IQR 0.7–3) and those with severe AKI (1.3 ng/ml, IQR 0.8–2.8, P = 0.99; “Fig 2B”). At the end of OLT (day 0), urinary NGAL concentrations increased to 18 ng/ml, IQR 1.6–56 in the no/mild AKI group (P<0.001) and to 20 ng/ml, IQR 3.2–62 in the severe AKI group (P = 0.009). Urinary NGAL concentrations at the end of OLT did not differ among groups (P = 0.98). At day 1, urine NGAL concentrations remained elevated in patients with no/mild AKI (15 ng/ml, IQR 6–36, P = 0.002) and in patients with severe AKI (59 ng/ml, IQR 27–90, P<0.001). Urine NGAL concentrations at day 1 were lower in patients with no/mild AKI than in patients with severe AKI (P = 0.002). At day 2 after OLT, urine NGAL concentrations returned to baseline values in the no/mild AKI group (5 ng/ml, IQR 1.8–13, P = 0.06 vs. baseline), but remained elevated in the severe AKI group (23 ng/ml, IQR 2–68, P = 0.01 vs. baseline).

### Performance of MIF and NGAL for predicting the development of AKI after OLT

**Fig 3:**
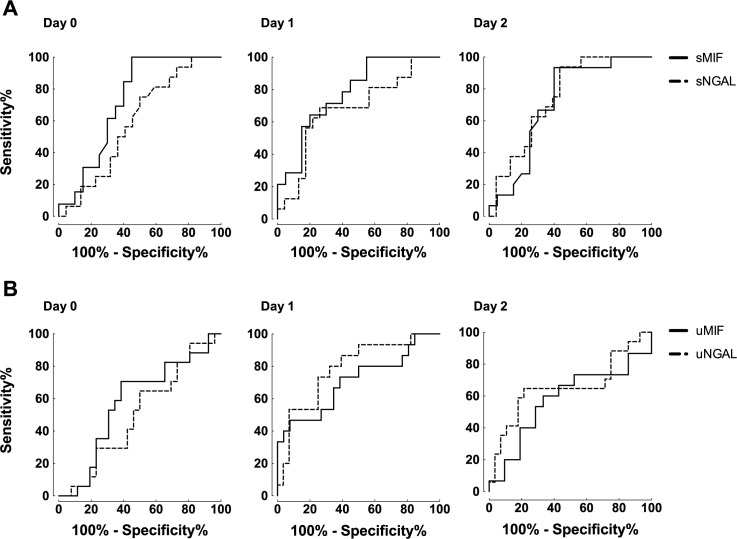
Receiver operating characteristic (ROC) curves of MIF and NGAL for predicting severe AKI after OLT. (A) ROC curves of serum MIF (sMIF, solid line) and serum NGAL (sNGAL, dashed line) at day 0, day 1 and day 2 after OLT. (B) ROC curves of urinary MIF (uMIF, solid line) and urinary NGAL (uNGAL, dashed line) at day 0, day 1 and day 2 after OLT.

In order to evaluate the predictive value of serum MIF, serum NGAL, urinary MIF, and urinary NGAL for the development of severe AKI after OLT, ROC curve analyses were performed. At the end of OLT, serum MIF was a fair predictor for severe AKI after OLT, whereas serum NGAL, urinary MIF, and urinary NGAL were not predictive for severe AKI (Table 3 and “Fig 3”). On day 1, serum MIF, urinary MIF, and urinary NGAL had fair predictive performance, whereas serum NGAL was a poor predictor for severe AKI. On day 2, serum MIF and serum NGAL had a fair value to diagnose severe AKI, whereas urinary NGAL had poor diagnostic value. Urinary MIF had no discriminatory power for detection of severe AKI at day 2.

**Table 3:** Area under the ROC curve for development of severe AKI.

	Serum MIF	Serum NGAL	Urine MIF	Urine NGAL
Day 0				
AUC	0.73	0.59	0.58	0.50
95% CI	0.55–0.90	0.41–0.77	0.40–0.75	0.33–0.68
P Value	0.03	0.34	0.40	0.98
Day 1				
AUC	0.78	0.68	0.71	0.79
95% CI	0.62–0.93	0.50–0.85	0.53–0.88	0.64–0.93
P Value	0.006	0.06	0.028	0.002
Day 2				
AUC	0.71	0.75	0.58	0.65
95% CI	0.54–0.89	0.60–0.90	0.37–0.78	0.47–0.84
P Value	0.03	0.009	0.43	0.08

Abbreviations: AKI, acute kidney injury; AUC, area under the curve; CI, confidence intervals; MIF, macrophage migration inhibitory factor; NGAL, neutrophil gelatinase-associated lipocalin-2.

Finally, we evaluated the predictive value of serum MIF, serum NGAL, urinary MIF, and urinary NGAL for the development of any stage of AKI after OLT. Serum MIF and serum NGAL did not predict the development of AKI at the end of OLT, but predicted AKI with good accuracy at day 1. Furthermore, serum MIF and serum NGAL had good value to diagnose AKI any stage at day 2 after OLT (Table 4). Urinary MIF and urinary NGAL did not adequately predict nor diagnose the development of AKI after OLT at any of the time points assessed.

**Table 4:** Area under the ROC curve for development of any stage of AKI.

	Serum MIF	Serum NGAL	Urine MIF	Urine NGAL
Day 0				
AUC	0.59	0.64	0.65	0.55
95% CI	0.36–0.83	0.44–0.85	0.51–0.78	0.38–0.66
P Value	0.48	0.23	0.05	0.62
Day 1				
AUC	0.81	0.82	0.73	0.59
95% CI	0.61–1	0.63–0.99	0.51–0.99	0.37–0.82
P Value	0.02	0.008	0.06	0.4
Day 2				
AUC	0.86	0.88	0.73	0.7
95% CI	0.73–0.99	0.77–1	0.45–1	0.57–0.93
P Value	0.006	0.002	0.12	0.06

Abbreviations: AKI, acute kidney injury; AUC, area under the curve; CI, confidence intervals; MIF, macrophage migration inhibitory factor; NGAL, neutrophil gelatinase-associated lipocalin-2.

## Discussion

In the current study, we serially measured serum and urine concentrations of MIF and NGAL in patients undergoing OLT, and compared the ability of these parameters to predict the development of severe AKI after OLT. After OLT, MIF concentrations in serum and urine were greater in patients who developed severe AKI than in those with normal postoperative kidney function. Macrophage migration inhibitory factor had equal power to predict severe AKI after OLT as NGAL, which is an established biomarker for AKI.

Clinical studies showed that NGAL was a good predictor of AKI after cardiac surgery [23], after out of hospital cardiac arrest [24], and in sepsis [25]. In patients undergoing OLT, two studies demonstrated that systemic NGAL was an early predictor of postoperative AKI [4,14]. Niemann et al. reported that a single measurement of plasma NGAL two hours after reperfusion during OLT predicted AKI in patients with a baseline sCr below 1.5 mg/dl [4], while Portal et al. stated that a single measurement of plasma NGAL within 24 hours after OLT predicted AKI with high accuracy [14]. Urinary NGAL was also proposed as an early marker for AKI after OLT. Two studies demonstrated that urinary NGAL concentrations measured within 24 hours after OLT could predict AKI after OLT [14,16], while Wagener et al. reported that urinary NGAL was able to predict AKI at 3 and 18 hours after OLT [15]. Our study confirmed the predictive value of urinary NGAL on day 1 after OLT, while serum NGAL only confirmed diagnosis of severe AKI on day 2 after OLT. Neither serum nor urinary NGAL had a predictive value for severe AKI at the end of surgery. These differences between our findings and previous studies might partially be due to the different timing of sample collection. In addition, the classification of AKI varied between studies. Previous studies classified AKI according to the Risk, Injury, Failure (RIFLE) criteria or the acute kidney injury network (AKIN) criteria, whereas we used the KDIGO criteria. Furthermore, not all authors differentiated between mild and severe AKI, but compared patients with AKI to those without AKI.

Macrophage migration inhibitory factor is a pro-inflammatory cytokine mediating the inflammatory response [26]. Elevated MIF concentrations lead to leukocyte chemotaxis and activation [27,28], resulting in histopathological damage of organs such as lungs [29] or kidneys [30]. Experimental studies have demonstrated the pathogenic role of MIF in immune-mediated renal injury [31,32], crescentic glomerulonephritis [33], and podocyte injury [34]. In humans, the role of MIF in kidney disease is less well described. Systemic MIF concentrations are elevated in patients with chronic kidney disease [35] and in septic patients with AKI [17]. Recently, we have demonstrated elevated plasma MIF concentrations in patients undergoing OLT and have suggested plasma MIF as a potential predictor for the requirement of renal replacement therapy after OLT [7]. Furthermore, urinary MIF has been proposed as a diagnostic tool in kidney disease. Urinary MIF concentrations were increased in patients with acute pyelonephritis, acute renal rejection, and proliferative glomerulonephritis, and correlated with renal MIF expression and the degree of renal injury [18–20]. Hong et al. suggested urinary MIF as a biomarker for acute pyelonephritis [19], whereas Brown et al. proposed that urine concentrations of MIF could be used to differentiate between acute transplant rejection and cyclosporine nephrotoxicity in renal transplant patients [20]. In the current study, we confirmed that serum MIF has predictive value for the development of severe AKI after OLT. In addition, our results suggest that urinary MIF might have a predictive value for severe AKI in the setting of OLT.

Our decision to focus on prediction of severe AKI was based on data from a previous investigation. Waikar et al. proposed that using small changes in creatinine to define AKI would lead to a high sensitivity but low specificity, and would significantly reduce the reliability of the novel biomarker [36]. Therefore, the investigators suggested using stages 2 and 3 of AKI to assess the predictive performance of novel biomarkers. In order to validate the performance of MIF as a novel biomarker for severe AKI, we compared its power to predict severe AKI to that of the well-established biomarker NGAL. Our results indicate that serum MIF has a fair predictive value for severe AKI after OLT on day 0, whereas the first time point at which urinary MIF and urinary NGAL had a fair predictive performance was on day 1. Due to the small number of patients these results can only be viewed as hypothesis-generating. We also assessed the predictive performance of MIF and NGAL for the development of AKI in general, i.e. stage 1, 2 or 3. The power of MIF and NGAL to predict any stage of AKI at the end of OLT was poor. At day 1 after OLT, serum MIF and serum NGAL had good predictive performance for AKI, as indicated by an AUC > 0.8 for both parameters. Urinary MIF and urinary NGAL did not predict the development of AKI at the end of OLT and on day 1 after OLT. Taken together, these results suggest that both serum MIF and serum NGAL have similar power to predict AKI after OLT. In contrast, urinary MIF and urinary NGAL only predict severe AKI after OLT. Furthermore, based on our results we speculate that serum MIF might be an earlier indicator for severe AKI after OLT than serum NGAL and urinary NGAL.

Another previously un-described finding of this study is the elevation of urine MIF concentrations in patients undergoing OLT. Urine MIF concentrations peak in all patients at the end of OLT, decrease on day 1, and return to baseline values on day 2. This finding might partially be explained by the renal clearance of systemic MIF, which was markedly elevated at the end of OLT. However, patients who developed severe AKI had greater urine MIF concentrations on day 1 after OLT than patients with normal kidney function. Previous studies have demonstrated that renal MIF is constitutively expressed in the normal kidneys and is upregulated in inflammatory kidney disease [18,37]. The upregulation of MIF correlated significantly with the concentration of urinary MIF in patients with glomerulonephritis, pyelonephritis and renal allograft rejection, while systemic MIF concentrations were not increased in these patients. The authors concluded that urinary MIF concentrations increase due to MIF production and secretion by the injured kidney, and proposed that urinary MIF might be a more specific parameter for kidney injury than systemic MIF [18–20]. In particular, Hong et al. suggested that urinary MIF, but not serum MIF, was an indicator for AKI in patients with acute pyelonephritis [19]. In contrast, we report elevated concentrations of serum and urinary MIF in patients developing AKI after OLT. Serum MIF predicted AKI after OLT earlier than urinary MIF. An explanation for these variable results might be different pathomechanisms in pyelonephritis-induced AKI and AKI after OLT. In pyelonephritis, AKI develops mainly due to a local infection ascending form the urogenital tract towards the kidneys, and thus is classified as renal or postrenal AKI. After OLT, the development of AKI is primarily based on prerenal causes including hypovolemia and hypotension, which result in renal hypoperfusion and ischemia.

Of note, there are limitations for the use of MIF as a biomarker for AKI, which are similar to those of using NGAL as a biomarker. A variety of human cells, including immune cells and hepatocytes, express MIF and NGAL [26,38]. Hence, any injury to those cells would increase systemic MIF and NGAL concentrations. In addition, MIF and NGAL are mediators of the innate immune response [39,40], and systemic concentrations of MIF and NGAL increase in patients with inflammatory diseases [41,42]. Therefore, the release of MIF and NGAL under various circumstances not primarily related to kidney injury could reduce the specificity of these proteins as biomarkers for early prediction of AKI. Furthermore, an overall limitation of this study is the small number of patients enrolled. In addition, the preoperative sCr of all included patients was below 1.5 mg/dl, suggesting absence of preexisting renal dysfunction. Thus, the question whether MIF can predict AKI in patients with preoperative renal dysfunction remains to be answered.

In conclusion, the results of this study suggest that serum MIF can predict AKI at an early postoperative stage after OLT. In the setting of OLT, MIF showed similar potency in predicting severe AKI after OLT as NGAL, and therefore might be useful as a novel biomarker for severe AKI.

## Supporting information

S1 Fig Perioperative serum creatinine concentrations at 4 different time points: baseline (BL; under anesthesia before skin incision), day 0 (at the end of surgery), day 1 (24 hours after graft reperfusion on day 1 after OLT), and on day 2 (48 hours after reperfusion day 2 after OLT). White bars indicate values of patients with no AKI or stage 1 AKI, gray bars represent values of patients who developed stage 2 or 3 AKI after undergoing OLT. P values indicate significant differences between groups.
(EPS)

## References

[1] Barri YM, Sanchez EQ, Jennings LW, Melton LB, Hays S, Levy MF, et al Acute kidney injury following liver transplantation: Definition and outcome. Liver Transpl. 2009 5;15(5):475–83. doi: 10.1002/lt.21682 19399734 10.1002/lt.21682

[2] Cabezuelo JB, Ramírez P, Ríos A, Acosta F, Torres D, Sansano T, et al Risk factors of acute renal failure after liver transplantation. Kidney Int Sup. 2006 2 1;69(6):1073–80. 10.1038/sj.ki.5000216 16528257

[3] Fraley DS, Burr R, Bernardini J, Angus D, Kramer DJ, Johnson JP. Impact of acute renal failure on mortality in end-stage liver disease with or without transplantation. Kidney Int Sup. 1998;54(2):518–24. 10.1046/j.1523-1755.1998.00004.x 9690218

[4] Niemann CU, Walia A, Waldman J, Davio M, Roberts JP, Hirose R, et al Acute kidney injury during liver transplantation as determined by neutrophil gelatinase-associated lipocalin. Liver Transpl. 2009 12;15(12):1852–60. doi: 10.1002/lt.21938 19938135 10.1002/lt.21938

[5] Kundakci A, Pirat A, Komurcu O, Torgay A, Karakayalı H, Arslan G, et al Rifle criteria for acute kidney dysfunction following liver transplantation: incidence and risk factors. TPS. 2010 12;42(10):4171–4. 10.1016/j.transproceed.2010.09.137 21168655

[6] Hilmi IA, Damian D, Al-Khafaji A, Planinsic R, Boucek C, Sakai T, et al Acute kidney injury following orthotopic liver transplantation: incidence, risk factors, and effects on patient and graft outcomes. Br J Anaesth. 2015 6;114(6):919–26. doi: 10.1093/bja/aeu556 25673576 10.1093/bja/aeu556

[7] Stefaniak J, Schiefer J, Miller EJ, Krenn CG, Baron DM, Faybik P. Macrophage migration inhibitory factor as a potential predictor for requirement of renal replacement therapy after orthotopic liver transplantation. Liver Transpl. 2015 4 24;21(5):662–9. doi: 10.1002/lt.24103 25762421 10.1002/lt.24103

[8] Utsumi M, Umeda Y, Sadamori H, Nagasaka T, Takaki A, Matsuda H, et al Risk factors for acute renal injury in living donor liver transplantation: evaluation of the RIFLE criteria. Transplant International. 2013 7 16;26(8):842–52. doi: 10.1111/tri.12138 23855657 10.1111/tri.12138

[9] Zarbock A, Kellum JA, Schmidt C, Van Aken H, Wempe C, Pavenstädt H, et al Effect of Early vs Delayed Initiation of Renal Replacement Therapy on Mortality in Critically Ill Patients With Acute Kidney Injury. JAMA. 2016 5 24;315(20):2190–9. 10.1001/jama.2016.5828 27209269

[10] Gaudry S, Hajage D, Schortgen F, Martin-Lefevre L, Pons B, Boulet E, et al Initiation Strategies for Renal-Replacement Therapy in the Intensive Care Unit. N Engl J Med. 2016 7 14;375(2):122–33. doi: 10.1056/NEJMoa1603017 27181456 10.1056/NEJMoa1603017

[11] Kidney Disease: Improving Global Outcomes (KDIGO) Acute Kidney Injury Work Group. KDIGO Clinical Practice Guideline for Acute Kidney Injury. Kidney Int Sup. 2012 2 7;2:1–138.

[12] Agarwal B. Difficulties in diagnosing acute kidney injury post liver transplantation using serum creatinine based diagnostic criteria. WJH. 2014;6(10):696–9. doi: 10.4254/wjh.v6.i10.696 25349641 10.4254/wjh.v6.i10.696 PMC4209415

[13] Bellomo R, Kellum JA, Ronco C. Defining acute renal failure: physiological principles. Intensive Care Med. 2004 1 1;30(1):33–7. doi: 10.1007/s00134-003-2078-3 14618231 10.1007/s00134-003-2078-3

[14] Portal AJ, McPhail MJW, Bruce M, Coltart I, Slack A, Sherwood R, et al Neutrophil gelatinase-Associated lipocalin predicts acute kidney injury in patients undergoing liver transplantation. Liver Transpl. 2010 10 28;16(11):1257–66. doi: 10.1002/lt.22158 21031541 10.1002/lt.22158

[15] Wagener G, Minhaz M, Mattis FA, Kim M, Emond JC, Lee HT. Urinary neutrophil gelatinase-associated lipocalin as a marker of acute kidney injury after orthotopic liver transplantation. Nephrol Dial Transplant. 2011 4 28;26(5):1717–23. doi: 10.1093/ndt/gfq770 21257679 10.1093/ndt/gfq770 PMC3145384

[16] Sirota JC, Walcher A, Faubel S, Jani A, McFann K, Devarajan P, et al Urine IL-18, NGAL, IL-8 and serum IL-8 are biomarkers of acute kidney injury following liver transplantation. BMC Nephrology. 2013 1 17;14(1):17. 23327592 10.1186/1471-2369-14-17 PMC3562144

[17] Payen D, Lukaszewicz A-C, Legrand M, Gayat E, Faivre V, Megarbane B, et al A Multicentre Study of Acute Kidney Injury in Severe Sepsis and Septic Shock: Association with Inflammatory Phenotype and HLA Genotype. PLoS ONE. 2012 6 6;7(6):e35838 doi: 10.1371/journal.pone.0035838 22701553 10.1371/journal.pone.0035838 PMC3368929

[18] Brown FG, Nikolic-Paterson DJ, Hill PA, Isbel NM, Dowling J, Metz CM, et al Urine macrophage migration inhibitory factor reflects the severity of renal injury in human glomerulonephritis. J Am Soc Nephrol. 2001 12 31;13 Suppl 1:S7–13. 11792756

[19] Hong M-Y, Tseng C-C, Chuang C-C, Chen C-L, Lin S-H, Lin C-F. Urinary Macrophage Migration Inhibitory Factor Serves as a Potential Biomarker for Acute Kidney Injury in Patients with Acute Pyelonephritis. Mediators Inflamm. 2012;2012(10):1–9. 10.1155/2012/381358 PMC3540913 23319831

[20] Brown FG, Nikolic-Paterson DJ, Chadban SJ, Dowling J, Jose M, Metz CN, et al Urine macrophage migration inhibitory factor concentrations as a diagnostic tool in human renal allograft rejection. Transplantation. 2001 6 26;71(12):1777–83. 11455258 10.1097/00007890-200106270-00013

[21] Levey AS, Coresh J, Balk E, Kausz AT, Levin A, Steffes MW, et al National Kidney Foundation practice guidelines for chronic kidney disease: evaluation, classification, and stratification. Ann Intern Med. 2003 7 14;139(2):137–47. 12859163 10.7326/0003-4819-139-2-200307150-00013

[22] Haase-Fielitz A, Bellomo R, Devarajan P, Story D, Matalanis G, Dragun D, et al Novel and conventional serum biomarkers predicting acute kidney injury in adult cardiac surgery—A prospective cohort study*. Crit Care Med. 2009 2;37(2):553–60. doi: 10.1097/CCM.0b013e318195846e 19114878 10.1097/CCM.0b013e318195846e

[23] Ho J, Tangri N, Komenda P, Kaushal A, Sood M, Brar R, et al Urinary, Plasma, and Serum Biomarkers' Utility for Predicting Acute Kidney Injury Associated With Cardiac Surgery in Adults: A Meta-analysis. Am J Kidney Dis. Elsevier; 2015 12;66(6):993–1005. doi: 10.1053/j.ajkd.2015.06.018 26253993 10.1053/j.ajkd.2015.06.018

[24] Park SO, Ahn JY, Lee YH, Kim YJ, Min YH, Ahn HC, et al Plasma neutrophil gelatinase-associated lipocalin as an early predicting biomarker of acute kidney injury and clinical outcomes after recovery of spontaneous circulation in out-of-hospital cardiac arrest patients. Resuscitation. European Resuscitation Council, American Heart Association, Inc., and International Liaison Committee on Resuscitation.~Published by Elsevier Ireland Ltd; 2016 4 1;101:84–90. doi: 10.1016/j.resuscitation.2016.01.005 26826562 10.1016/j.resuscitation.2016.01.005

[25] Zhang A, Cai Y, Wang P-F, Qu J-N, Luo Z-C, Chen X-D, et al Diagnosis and prognosis of neutrophil gelatinase-associated lipocalin for acute kidney injury with sepsis: a systematic review and meta-analysis. Crit Care. BioMed Central; 2016 Feb 16;20(1):41. 26880194 10.1186/s13054-016-1212-x PMC4754917

[26] Calandra T, Roger T. Macrophage migration inhibitory factor: a regulator of innate immunity. Nat Rev Immunol. 2003 10;3(10):791–800. doi: 10.1038/nri1200 14502271 10.1038/nri1200 PMC7097468

[27] Bernhagen J, Krohn R, Lue H, Gregory JL, Zernecke A, Koenen RR, et al MIF is a noncognate ligand of CXC chemokine receptors in inflammatory and atherogenic cell recruitment. Nat Med. 2007 4 15;13(5):587–96. doi: 10.1038/nm1567 17435771 10.1038/nm1567

[28] Takahashi K, Koga K, Linge HM, Zhang Y, Lin X, Metz CN, et al Macrophage CD74 contributes to MIF-induced pulmonary inflammation. Respir Res. 2009;10(1):33. 19413900 10.1186/1465-9921-10-33 PMC2681459

[29] Lai KN, Leung JC, Metz CN, Lai FM, Bucala R, Lan HY. Role for macrophage migration inhibitory factor in acute respiratory distress syndrome. J Pathol. 2003;199(4):496–508. doi: 10.1002/path.1291 12635141 10.1002/path.1291

[30] Lan HY, Bacher M, Yang N, Mu W, Nikolic-Paterson DJ, Metz C, et al The pathogenic role of macrophage migration inhibitory factor in immunologically induced kidney disease in the rat. J Exp Med. 1997 4 20;185(8):1455–65. 9126926 10.1084/jem.185.8.1455 PMC2196273

[31] Pacheco EG, Silva ODCE, Sankarankutty AK, Ribeiro MAF. Analysis of the Liver Effluent as a Marker of Preservation Injury and Early Graft Performance. TPS. 2010 3 1;42(2):435–9. 10.1016/j.transproceed.2010.01.018 20304158

[32] Suehiro T, Boros P, Emre S, Sheiner P, Guy S, Schwartz M, et al Value of caval effluent in predicting early graft function after orthotopic liver transplantation. TPS. 1997 2;29(1–2):469–70. 10.1016/s0041-1345(96)00207-2 9123086

[33] Yang N, Nikolic-Paterson DJ, Ng Y, Mu W, Metz C, Bacher M, et al Reversal of established rat crescentic glomerulonephritis by blockade of macrophage migration inhibitory factor (MIF): potential role of MIF in regulating glucocorticoid production. Mol Med. 1998 5 31;4(6):413–24. 10780884 PMC2230272

[34] Sasaki S, Nishihira J, Ishibashi T, Yamasaki Y, Obikane K, Echigoya M, et al Transgene of MIF induces podocyte injury and progressive mesangial sclerosis in the mouse kidney. Kidney Int Sup. 2004 1 31;65(2):469–81. 10.1111/j.1523-1755.2004.00394.x 14717917

[35] Bruchfeld A, Carrero JJ, Qureshi AR, Lindholm B, Barany P, Heimburger O, et al Elevated serum macrophage migration inhibitory factor (MIF) concentrations in chronic kidney disease (CKD) are associated with markers of oxidative stress and endothelial activation. Mol Med. 2009 2 28;15(3–4):70–5. doi: 10.2119/molmed.2008.00109 19081768 10.2119/molmed.2008.00109 PMC2600496

[36] Waikar SS, Betensky RA, Emerson SC, Bonventre JV. Imperfect Gold Standards for Kidney Injury Biomarker Evaluation. J Am Soc Nephrol. 2012 1 6;23(1):13–21. doi: 10.1681/ASN.2010111124 22021710 10.1681/ASN.2010111124 PMC3695762

[37] Lan HY, Yang N, Nikolic-Paterson DJ, Yu XQ, Mu W, Isbel NM, et al Expression of macrophage migration inhibitory factor in human glomerulonephritis. Kidney Int Sup. 2000;57(2):499–509. 10.1046/j.1523-1755.2000.00869.x 10652026

[38] Schmidt-Ott KM, Mori K, Li JY, Kalandadze A, Cohen DJ, Devarajan P, et al Dual action of neutrophil gelatinase-associated lipocalin. J Am Soc Nephrol. American Society of Nephrology; 2007 2;18(2):407–13. doi: 10.1681/ASN.2006080882 17229907 10.1681/ASN.2006080882

[39] Calandra T, Bernhagen J, Metz CN, Spiegel LA, Bacher M, Donnelly T, et al MIF as a glucocorticoid-induced modulator of cytokine production. Nature. 1995 9 7;377(6544):68–71. doi: 10.1038/377068a0 7659164 10.1038/377068a0

[40] Flo TH, Smith KD, Sato S, Rodriguez DJ, Holmes MA, Strong RK, et al Lipocalin 2 mediates an innate immune response to bacterial infection by sequestrating iron. Nature. 2004 12 16;432(7019):917–21. doi: 10.1038/nature03104 15531878 10.1038/nature03104

[41] Leaver SK, MacCallum NS, Pingle V, Hacking MB, Quinlan GJ, Evans TW, et al Increased plasma thioredoxin levels in patients with sepsis: positive association with macrophage migration inhibitory factor. Intensive Care Med. 2009 9 15;36(2):336–41. doi: 10.1007/s00134-009-1640-z 19756498 10.1007/s00134-009-1640-z PMC2809307

[42] Lindberg S, Jensen JS, Mogelvang R, Pedersen SH, Galatius S, Flyvbjerg A, et al Plasma neutrophil gelatinase-associated lipocalinin in the general population: association with inflammation and prognosis. Arterioscler Thromb Vasc Biol. 2014 9;34(9):2135–42. doi: 10.1161/ATVBAHA.114.303950 24969771 10.1161/ATVBAHA.114.303950

